# Melatonin Receptor 1 and Melatonin Receptor 2 Expression During Human Kidney Development and Their Association with CAKUT

**DOI:** 10.3390/jdb14020018

**Published:** 2026-04-15

**Authors:** Ann-Kathrin Schmitt, Victoria Tjora, Nela Kelam, Marija Jurić Gunjača, Petar Todorović, Clelia Picard, Manel Loche-Dalmon, Katarina Vukojević, Anita Racetin

**Affiliations:** 1 Laboratory for Early Human Development, Department of Anatomy, Histology and Embryology, School of Medicine, University of Split, Šoltanska 2A, 21000 Split, Croatia; annkathrin.schmidt@mefst.hr (A.-K.S.); victoria.tjora@mefst.hr (V.T.); nela.kelam@mefst.hr (N.K.); petar.todorovic@mefst.hr (P.T.); katarina.vukojevic@mefst.hr (K.V.); 2 Center for Translational Research in Biomedicine, School of Medicine, University of Split, Šoltanska 2A, 21000 Split, Croatia; 3 Department of Radiology, General Hospital of Šibenik-Knin County, 22000 Sibenik, Croatia; marija.juric@mefst.hr; 4 Faculty of Medicine and Health Sciences, University of Angers, 49100 Angers, France; clelia.picard@etud.univ-angers.fr (C.P.); manel.loche-dalmon@etud.univ-angers.fr (M.L.-D.); 5 Mediterranean Institute for Life Sciences (MedILS), University of Split, Meštrovićevo Šetalište 45, 21000 Split, Croatia

**Keywords:** kidney development, CAKUT, melatonin, MTNR1A, MTNR1B

## Abstract

Background/Objectives: Growing evidence indicates that melatonin contributes to kidney development and function, while disruptions of fetal circadian signaling have been linked to congenital anomalies of the kidney and urinary tract (CAKUT). This study aimed to characterize the developmental and spatial expression patterns of melatonin receptors MTNR1A and MTNR1B in normal human fetal kidneys and in CAKUT phenotypes. Methods: This study analyzed 40 human fetal kidney specimens, including healthy controls and CAKUT cases (horseshoe kidneys, duplex kidneys, and dysplastic kidneys), obtained from spontaneous abortions and pregnancy terminations. Samples were classified into developmental phases Ph2–Ph4 according to established morphological criteria. Immunofluorescence staining was used to visualize MTNR1A and MTNR1B expression. Quantitative analysis was performed using ImageJ, measuring the fluorescence area percentage. Statistical comparisons were conducted using a two-way ANOVA. Results: In control kidneys, MTNR1A expression was predominantly observed in glomeruli and interstitial cells and showed a descending trend across developmental stages, whereas MTNR1B was localized to glomeruli and strongly to the apical membranes of tubules, particularly distal tubules, without substantial developmental variation. CAKUT phenotypes exhibited higher expression of both receptors compared to controls. Significant phase-dependent differences in MTNR1A expression were observed in horseshoe, duplex, and dysplastic kidneys. MTNR1B expression decreased across developmental stages in dysplastic kidneys and differed significantly between Ph3 and Ph4 in duplex kidneys. At Ph3, duplex kidneys showed the highest MTNR1B expression. Conclusions: Altered developmental expression patterns of MTNR1A and MTNR1B in CAKUT suggest an association between melatonin signaling and abnormal human kidney development.

## 1. Introduction

Melatonin is a pleiotropic hormone primarily secreted by the pineal gland at night and plays a crucial role in regulating circadian rhythms. Beyond its chronobiotic function, melatonin exhibits potent antioxidant, anti-inflammatory, anticancer, anti-apoptotic, antihypertensive, and immunoregulatory properties. Its role in human health spans the entire lifespan, with increasing evidence highlighting its significance in normal pregnancy and fetal development [1].

Melatonin exerts its effects primarily through melatonin receptors MTNR1A and MTNR1B, which are transmembrane G protein-coupled receptors, as well as through nuclear “orphan” receptors from the RORα/RZR family [2]. Furthermore, melatonin exerts receptor-independent effects by neutralizing reactive oxygen and nitrogen species [3]. The study of the peripheral distribution of melatonin receptors is crucial, as they represent attractive targets for immunomodulation, regulation of endocrine, reproductive, and cardiovascular functions, modulation of skin pigmentation and hair growth, and modulation of carcinogenesis and aging [2]. MTNR1A and MTNR1B receptors are expressed in various tissues, including the kidneys [4,5,6,7,8,9,10]. Drew et al. also reported the presence of MTNR1A and MTNR1B receptors in the human fetal kidney cortex [11]. Previous studies have shown that disruption of the fetal circadian rhythm may result in phenotypic changes resembling CAKUT [12]. Maternal melatonin deficiency has been associated with increased blood pressure in offspring, a common comorbidity of CKD [13]. Studies have shown that alterations in photoperiod during pregnancy negatively affect not only blood pressure but also renal function in adult rat offspring [14]. Thus, melatonin has been recognized as a potential therapeutic agent for the management of various kidney-related diseases, including hypertension [15], diabetes [16], acute kidney injury [17], chronic kidney disease (CKD) [18], and renal carcinoma [19].

Given the existing evidence on the potential role of melatonin in kidney development and function, we found it compelling to further investigate this signaling pathway. Thus, this study aimed to visualize the distribution of melatonin receptors MTNR1A and MTNR1B during normal human kidney development and in cases of CAKUT using immunofluorescence, to determine whether alterations in melatonin receptor distribution occur in congenital anomalies of the kidney and urinary tract.

## 2. Materials and Methods

### 2.1. Tissue Collection and Preparation

This study included 40 human fetal kidney specimens and CAKUT cases (duplex kidneys, horseshoe kidneys, and dysplastic kidneys), as shown in Table 1, derived from spontaneous abortions and pregnancy terminations performed due to severe congenital malformations. Samples were obtained from the Department of Pathology at University Hospital Split. All procedures involving tissue handling adhered to the Declaration of Helsinki guidelines and were approved by the Institutional Ethical and Drug Committee (reference: 520-03/25-01/78; approval number: 2181-147/01-06/LJ.Z.-25-02). Gestational age was estimated from external measurements and menstrual data, while kidney abnormalities were classified by gross morphological assessment and standard histopathological examination.

Kidney tissue samples were fixed in 4% paraformaldehyde. Following dehydration through an ascending alcohol gradient, the tissue was embedded in paraffin. Sections of 5 µm thickness were prepared using a microtome.

### 2.2. Separation of Samples Based on Developmental Phases

Development of the metanephros is classified into four phases based on the ureteric bud’s branching pattern and nephron-inducing capacity. Phase 1 (5th–14th gestational week) is characterized by active branching, with each ureteric bud tip forming a single nephron. During Phase 2 (15th–20th/22nd week), branching ceases, and each tip gives rise to multiple nephrons arranged in arcades. In Phase 3, which extends until the 36th week, the majority of nephrons are formed, with multiple nephrons attaching individually along the length of the collecting duct. In contrast, in Phase 4 (from the 36th week onward), ureteric bud tips disappear, nephron formation ends, and existing nephrons continue to mature [20,21,22,23]. In the present study, samples were categorized according to this established classification, and analyses focused on Phases 2, 3, and 4, while Phase 1 was excluded due to the considerable difficulty in obtaining specimens at such early developmental stages.

### 2.3. Immunofluorescence Staining

Paraffin-embedded tissue sections underwent deparaffinization in xylene followed by rehydration through a graded alcohol series. Antigen unmasking was performed by heating the sections in 0.01 M citrate buffer (pH 6.0). Following three washes with PBS, non-specific binding was blocked using a commercial blocking solution (ab64226, Abcam, Cambridge, UK) for 20 min. Upon removal of the blocking agent, primary antibodies were applied and left to incubate overnight in a humidified chamber (MTNR1A, ab203038, Abcam, Cambridge, UK, 1:100; and MTNR1B, AB203346, Abcam, Cambridge, UK, 1:100). The following day, sections were rinsed in PBS and subsequently exposed to secondary antibodies for 1 h at room temperature (Anti-Rabbit lgG, Alexa Fluor^®^488, 711-545-152, Jackson ImmunoResearch Laboratories, Inc., Baltimore, MD, USA, 1:300). After PBS washes, nuclear counterstaining was performed using DAPI (4′,6-diamidino-2-phenylindole). Sections were then mounted with coverslips using Immuno-Mount medium (Thermo Shandon, Pittsburgh, PA, USA). To verify staining specificity, negative control samples were processed identically but with the omission of the primary antibody.

### 2.4. Data Acquisition

Tissue sections from human fetal kidneys were imaged using an Olympus BX51 epifluorescence microscope (Olympus, Tokyo, Japan) equipped with a Nikon DS-Ri2 digital camera (Nikon Corporation, Tokyo, Japan). Image acquisition was performed using NIS-Elements F software (version 5.22.00). For each specimen, MTNR1A and MTNR1B expression were evaluated across 10 distinct fields within the cortex of healthy fetal kidneys. In CAKUT-affected specimens, particularly those exhibiting dysplastic features (DYSs), the architectural distortion made a reliable differentiation between cortex and medulla difficult, so we used 10 randomly chosen fields. The 10 fields per specimen were obtained from 2 to 3 non-serial tissue sections to ensure adequate sampling across the specimen. Prior to statistical analysis, fluorescence area percentage values from all fields belonging to the same specimen were averaged, so that the individual specimen, rather than the individual field, served as the unit of analysis. All images were acquired at 40× magnification using consistent exposure settings. Immunoreactivity for MTNR1A and MTNR1B appeared as either a diffuse or punctate green fluorescent signal. Quantitative analysis of the acquired images was conducted in ImageJ (version 1.54; National Institutes of Health, Bethesda, MD, USA), following previously established protocols [21,23,24]. Quantification of fluorescence area percentage was performed with the “Analyze Particles” function. To minimize inter-observer bias, threshold values were established using negative control images, and three experienced histologists independently assessed microphotographs. Reproducibility was validated by an intraclass correlation coefficient exceeding 0.8, reflecting excellent inter-rater agreement [25].

### 2.5. Statistical Analysis

All statistical analyses were performed using GraphPad Prism software (version 8.4.3, GraphPad Software, La Jolla, CA, USA), with statistical significance defined as *p* < 0.05. Data are presented as mean ± standard deviation (SD) from at least 3 biological replicates per group, with at least 10 representative microscopic fields analyzed per sample.

Data distribution normality was evaluated using the Shapiro–Wilk test, and all data were confirmed to be normally distributed. Expression levels of MTNR1A and MTNR1B were compared across developmental phases (Phases 2, 3, and 4) and between tissue groups (control and CAKUT-affected specimens) using two-way ANOVA followed by Tukey’s multiple comparison test. CAKUT phenotype combinations for which no specimens were available were excluded from the analysis and are consequently not represented in the figures (HK Ph4 and DK Ph2). Interaction effects between developmental phase and tissue type were evaluated.

Graphical representations were generated in GraphPad Prism 8.4.3, and the final figure assembly was completed in Adobe Photoshop version 9.0 (Adobe, San Jose, CA, USA). For optimal visual clarity, microphotographs were processed with background subtraction and contrast adjustment.

## 3. Results

### 3.1. Expression of MTNR1A

**Figure 1:**
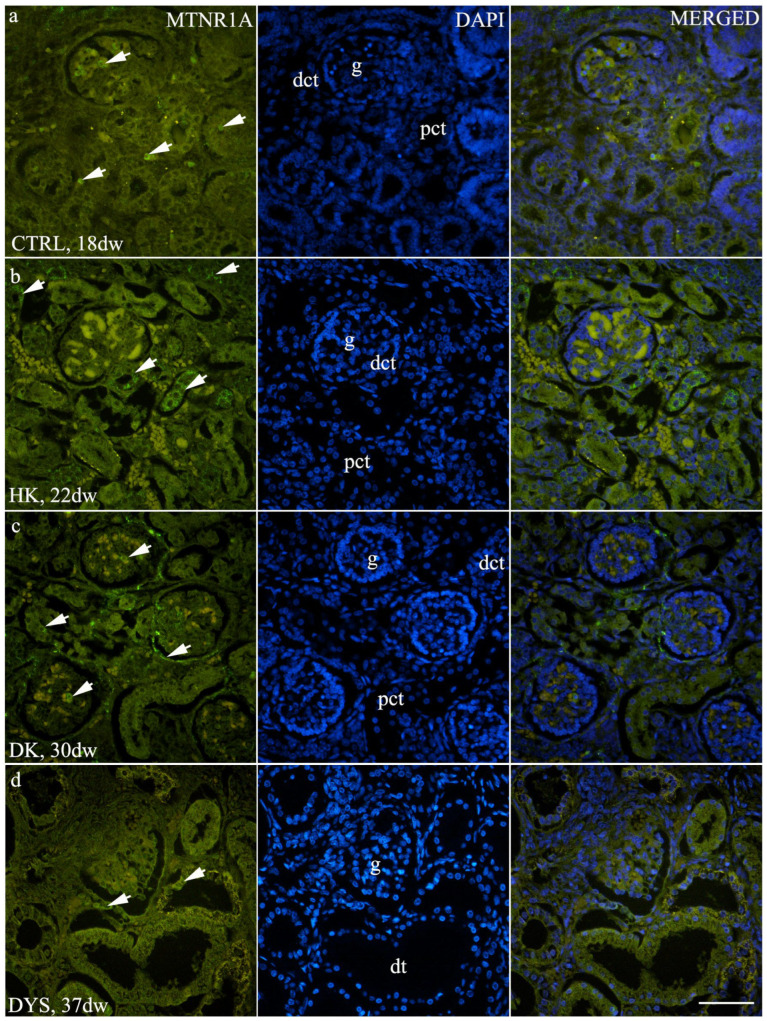
Immunofluorescence staining of (**a**) control human fetal kidneys (CTRL), (**b**) horseshoe kidneys (HK), (**c**) duplex kidneys (DK), and (**d**) dysplastic kidneys (DYS) using an antibody against melatonin receptor 1A (MTNR1A). Arrows indicate MTNR1A staining in the glomeruli (g), distal convoluted tubules (dct), dysplastic tubules (dt), and mild staining in the proximal convoluted tubules (pct), as indicated in the corresponding 4′,6-diamidino-2-phenylindole (DAPI)-stained images. All images were acquired at 40× magnification; scale bar is 50 µm.

**Figure 2:**
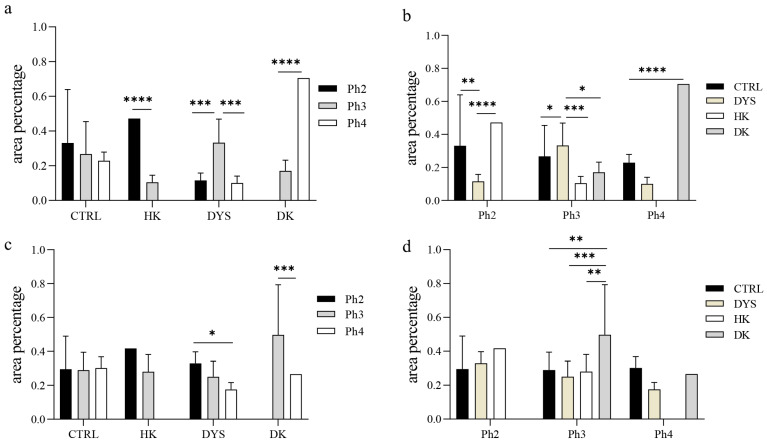
Graphs showing the area percentages of MTNR1A (**a**,**b**) and MTNR1B (**c**,**d**) in control (CTRL) and CAKUT tissues across developmental Phases 2, 3, and 4 (**a**,**c**) and between different groups within the same developmental phase (**b**,**d**). CAKUT tissues were subdivided into dysplastic kidneys (DYS), horseshoe kidneys (HK), and duplex kidneys (DK). CAKUT phenotype combinations for which no specimens were available are not displayed (HK Ph4 and DK Ph2). Data are presented as mean ± standard deviation (SD) and were analyzed using a two-way ANOVA. Statistically significant differences are indicated as * *p* < 0.05, ** *p* < 0.01, *** *p* < 0.001, and **** *p* < 0.0001. Ten representative images were analyzed per sample.

In the healthy human kidney (control) group, prominent MTNR1A expression was observed in the glomeruli, as well as in some cells of the interstitium (Figure 1a). Mild tubular staining was occasionally present. Two-way ANOVA revealed a significant main effect of tissue group (F (3,84) = 5.641, *p* = 0.0014) and a significant interaction (F (6,84) = 42.89, *p* < 0.0001), while the main effect of developmental phase was not significant (F (2,84) = 1.025, *p* = 0.3632). Although no statistically significant differences were detected among developmental stages within the control group, a descending trend in MTNR1A area percentage was observed, with more mature developmental stages showing lower expression levels (Figure 2a).

Horseshoe kidneys (HK) and duplex kidneys (DK) showed prominent MTNR1A expression. In HK samples, strong expression was observed in the tubular compartment, particularly in the distal tubules (Figure 1b). In DK samples, marked expression was detected in the distal tubules and was even more pronounced in the glomeruli, especially in the parietal layer of Bowman’s capsule (Figure 1c). Due to the limited availability of samples across all developmental phases, a continuous trend of expression could not be assessed in the HK and DK groups. However, a statistically significant difference in MTNR1A area percentage was observed between Ph2 and Ph3 in the HK group (Figure 2a, *p* < 0.0001), as well as between Ph3 and Ph4 in the DK group (Figure 2a, *p* < 0.0001). In the dysplastic kidney group, mild MTNR1A expression was observed in the glomeruli and dysplastic tubules (dt) (Figure 1d) during Ph2 and Ph4, while a statistically significant increase in area percentage was detected during Ph3 (Figure 2a, *p* < 0.001).

By comparing MTNR1A expression between different phenotypes within the same developmental phase, distinct differences were observed. At the developmental stage Ph2, horseshoe kidneys showed significantly higher MTNR1A expression than in all other groups (Figure 2b, *p* < 0.0001). In contrast, at developmental stage Ph3, dysplastic kidneys exhibited the most prominent MTNR1A expression among all analyzed phenotypes (Figure 2b, *p* < 0.001). At Ph4, duplex kidneys demonstrated the highest area percentage of MTNR1A expression (*p* < 0.0001). Overall, MTNR1A expression was consistently higher in CAKUT phenotypes compared with control samples across all analyzed developmental stages.

### 3.2. Expression of MTNR1B

**Figure 3:**
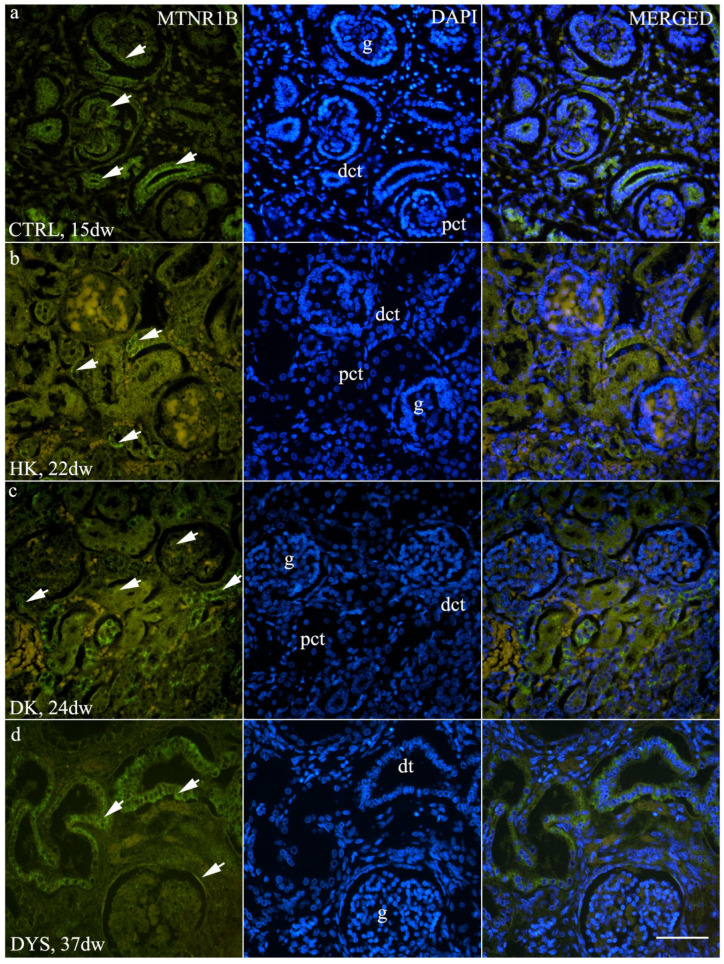
Immunofluorescence staining of (**a**) control human fetal kidneys (CTRL), (**b**) horseshoe kidneys (HK), (**c**) duplex kidneys (DK), and (**d**) dysplastic kidneys (DYS) using an antibody against melatonin receptor 1B (MTNR1B). Arrows indicate MTNR1B staining in the glomeruli (g), distal convoluted tubules (dct), dysplastic tubules (dt), and mild staining in the proximal convoluted tubules (pct), as indicated in the corresponding 4′,6-diamidino-2-phenylindole (DAPI)-stained images. All images were acquired at 40× magnification; scale bar is 50 µm.

In the control group, MTNR1B expression was detected in the glomeruli and was strongly localized to the apical membranes of the tubules, particularly in the distal tubules (Figure 3a). Two-way ANOVA revealed a significant main effect of developmental phase (F (2,84) = 11.93, *p* < 0.0001) and a significant interaction (F (6,84) = 17.85, *p* < 0.0001), while the main effect of tissue group did not reach statistical significance (F (3,84) = 1.218, *p* = 0.3083). No substantial variation in MTNR1B expression remained relatively stable across developmental stages within the control group (Figure 2c, *p* > 0.05).

Horseshoe kidneys (HK) and duplex kidneys (DK) exhibited a very similar MTNR1B expression pattern. Strong expression was observed in the tubular compartment, predominantly in the distal tubules, but also in the proximal tubules, with mild expression detected in the glomeruli (Figure 3b,c). Due to the limited availability of samples across all developmental stages, a continuous expression trend could not be evaluated in the HK and DK groups. However, a statistically significant difference in MTNR1B area percentage was observed between developmental stages Ph3 and Ph4 in the DK group (Figure 2c, *p* < 0.001). In the dysplastic kidney (DYS) group, strong MTNR1B expression was observed in the dysplastic tubules and the parietal layer of Bowman’s capsule (Figure 3d). A statistically significant descending trend in MTNR1B area percentage was detected across developmental stages within the DYS group, with more mature stages exhibiting lower expression levels (Figure 2c, *p* < 0.05).

Comparison of MTNR1B expression between different phenotypes within the same developmental stage revealed significant differences at developmental stage Ph3. Duplex kidneys demonstrated significantly higher MTNR1B expression compared to all other groups (Figure 2d, *p* < 0.001).

## 4. Discussion

Melatonin plays an important role in human health throughout the lifespan, with increasing evidence highlighting its significance in normal pregnancy and fetal development [1]. Its physiological effects are pleiotropic and involve multiple organ systems, supporting the concept of melatonin as a potential therapeutic agent in various diseases across different age groups. Melatonin acts predominantly through the membrane receptors MTNR1A and MTNR1B, while additional receptor-independent effects, including antioxidant activity and interactions with intracellular proteins, have also been described [3,6]. Given that melatonin receptors represent potential targets for immunomodulation and broader physiological regulation, understanding their peripheral distribution is of particular importance [2]. This rationale motivated our investigation of melatonin receptor distribution during normal human kidney development.

Our results demonstrated prominent expression of both MTNR1A and MTNR1B during developmental phases 2–4, with localization in glomeruli, the parietal layer of Bowman’s capsule, and tubular structures, particularly distal tubules. Receptor expression remained relatively stable across the analyzed developmental stages. These findings are consistent with previous studies reporting widespread expression of MTNR1A and MTNR1B in multiple tissues, including the kidney [4,5,6,7,8,9]. Notably, the presence of both receptors in the human fetal kidney cortex has been previously described [11], further supporting a role for melatonin signaling in renal development. In CAKUT samples, a similar expression pattern was observed, with prominent localization in glomeruli and tubules, especially distal and dysplastic tubules, suggesting that circadian signaling may contribute to normal and abnormal kidney development. This is in line with earlier reports showing that disruption of the fetal circadian rhythm can result in phenotypic changes resembling CAKUT [12]. Both receptors signal predominantly through Gi-coupled pathways, leading to inhibition of cAMP production and activation of downstream effectors, including ERK/MAPK and PI3K/Akt [26,27]. Notably, these pathways intersect with Wnt/β-catenin signaling, which is essential for ureteric bud branching and nephron progenitor maintenance during kidney development, and its dysregulation has been implicated in CAKUT pathogenesis [28,29].

Experimental evidence further supports the developmental relevance of melatonin. Altered photoperiod exposure and maternal melatonin supplementation during pregnancy have been shown to induce transcriptomic and epigenetic changes in offspring kidneys, particularly during nephrogenesis [30,31]. Moreover, maternal melatonin deficiency has been associated with increased blood pressure in offspring, a frequent comorbidity of chronic kidney disease [13,14]. Consequently, melatonin has been proposed as a potential therapeutic agent in several kidney-related conditions, including hypertension, diabetes, acute kidney injury, chronic kidney disease, and renal carcinoma [15,16,17,18,19].

Certain limitations of this study should be considered when interpreting the results. The relatively small number of available samples, unequal representation of developmental stages and CAKUT phenotypes, and the absence of complementary methodological approaches beyond immunohistochemistry limit the ability to draw functional conclusions. Furthermore, the time of sample collection was not recorded; given that melatonin receptor expression may be subject to circadian variation, this represents an additional source of variability that cannot be excluded. Finally, the observed differences in receptor localization may partially reflect morphological redistribution associated with structural remodeling rather than altered expression per se. Nevertheless, given the rarity, sensitivity, and high biological value of human fetal kidney samples, the data presented here provide a descriptive foundation for future studies employing larger cohorts and complementary experimental techniques.

Despite the growing body of evidence supporting the beneficial effects of melatonin, its molecular and cellular mechanisms in kidney development and disease remain incompletely understood. Further studies investigating the specific roles of MTNR1A and MTNR1B, both individually and in combination, as well as the effects of exogenous melatonin administration, are warranted. Such approaches may help clarify receptor-specific roles in nephrogenesis and support the development of targeted strategies aimed at preserving kidney function.

## Figures and Tables

**Table 1:** The samples of human embryonic and fetal kidneys analyzed in the study.

Groups	Developmental Phase	Renal and Associated Pathology	Gestational Phases (dw)	Total Number of Kidney Samples
	Ph2	N/A	15	6
		N/A	16	
		N/A	17	
		N/A	18	
		N/A	21	
	Ph3	N/A	23	6
		N/A	24	
		N/A	28	
		N/A	29	
	Ph4	N/A	32	6
		N/A	35	
		N/A	37	
		N/A	38	
Horseshoe kidney (HK)	Ph2	*Ren concreatus arcuatus*, *cystae multiplices corticales*	22	1
	Ph3	*Ren concreatus arcuatus*, *tetras Fallot*	26	4
		*Syndroma Edwards*, *Ren arcuatus*	30–31	
		*Syndroma Edwards*, *Ren arcuatus*	34	
Dysplastic kidneys (DYS)	Ph2	*Megaureter lateris dextri*, *Dysplasia renis*	21	3
		*Renis dysplastica cysticus lateralis sinistri*, *agenesia renis dextri*		
	Ph3	*Dysplasia multicystica renis dextri*	27	5
		*Renes dysplastici cystici*, *Syndroma Potter*	35	
		*Dysplasia renis multicystica bilateralis*		
	Ph4	*Agenesis renis dextri et dysplasia renis sinistri cum ureter duplex*	37	5
		*Dysplasia hypoplastica*, *renis bilateralis*, *syndroma Down, syndroma Potter*	38	
		*Syndroma Potter*, *Dysplasia renis*	39	
Duplex kidneys (DK)	Ph3	*Ureter duplex lateris dextri*	24	3
		*Ureter duplex lateris sinistri*	30	
	Ph4	*Pyelon et ureter duplex bilateralis*	41	1

## Data Availability

The original contributions presented in this study are included in the article. Further inquiries can be directed to the corresponding author.

## Abbreviations

The following abbreviations are used in this manuscript:

ANOVA	Analysis of variance
CAKUT	Congenital anomalies of the kidney and urinary tract
CKD	Chronic kidney disease
CTRL	Control
DAPI	4′,6-diamidino-2-phenylindole
dct	Distal convoluted tubule
DK	Duplex kidneys
dt	dysplastic tubules
DYS	Dysplastic kidneys
g	glomeruli
HK	Horseshoe kidneys
MTNR1A	Melatonin receptor 1A
MTNR1B	Melatonin receptor 1B
PBS	Phosphate-buffered saline
pct	Proximal convoluted tubules
Ph2	Phase 2
Ph3	Phase 3
Ph4	Phase 4
SD	Standard deviation
